# Work: saviour or struggle? A qualitative study examining employment and finances in colorectal cancer survivors living with advanced cancer

**DOI:** 10.1007/s00520-022-07307-9

**Published:** 2022-08-16

**Authors:** Chloe Yi Shing Lim, Rebekah C. Laidsaar-Powell, Jane M. Young, Daniel Steffens, Bogda Koczwara, Yuehan Zhang, The advanced-CRC survivorship authorship group, Chloe Yi Shing Lim, Rebekah C. Laidsaar-Powell, Jane M. Young, Michael Solomon, Daniel Steffens, Cherry Koh, Nabila Ansari, David Yeo, Prunella Blinman, Philip Beale, Bogda Koczwara, Grace Joshy, Yuehan Zhang, Phyllis Butow

**Affiliations:** 1 Centre for Medical Psychology and Evidence-Based Decision-Making (CeMPED), School of Psychology, Faculty of Science, The University of Sydney, Sydney, NSW Australia; 2 Sydney School of Public Health, Faculty of Medicine and Health, The University of Sydney, Sydney, NSW Australia; 3 The Daffodil Centre, The University of Sydney, a joint venture with Cancer Council NSW, Sydney, Australia; 4 Surgical Outcomes Research Centre (SOuRCe), Sydney Local Health District, Sydney, NSW Australia; 5 Faculty of Medicine and Health, Central Clinical School, The University of Sydney, Sydney, NSW Australia; 6 Department of Medical Oncology, Flinders Medical Center, Bedford Park, South Australia Australia; 7 National Breast Cancer Foundation, Sydney, NSW Australia; 8 National Centre for Epidemiology and Population Health, Research School of Population Health, Australian National University, Canberra, ACT Australia

**Keywords:** Bowel cancer, Cancer survivorship, Return to work, Financial wellbeing, Qualitative interviews

## Abstract

**Purpose:**

Continuing employment or returning to work (RTW) as a cancer survivor can be meaningful and financially necessary, yet challenging. However, there is a lack of qualitative research on RTW experiences and financial wellbeing of people with advanced colorectal cancer (CRC-A). This study aimed to fill this gap.

**Methods:**

Adults treated for CRC-A were recruited 0.5–2 years post-surgery (or post-diagnosis of CRC-A for palliative chemotherapy participants). Semi-structured telephone interviews, exploring RTW and finances, were subjected to framework analysis. Demographic, clinical, and quality of life data (FACT-C, Distress Thermometer, COST measure) were collected to characterise the sample and inform the framework analysis.

**Results:**

Analysis of 38 interviews revealed five overarching themes: *work as a struggle*, *work as my identity*, *work as my saviour*, *work as a financial necessity*, and *employer and colleague response.* Many survivors with CRC-A desired to, and had the capacity to, continue work or RTW, yet faced unique challenges from compounded stigma of both cancer and toileting issues. Inability to RTW negatively impacted financial and psychosocial wellbeing. Workplace support was an important facilitator of RTW.

**Conclusion:**

For survivors with CRC-A, continuing or RTW is fraught with challenges, including physical functioning challenges, financial anxiety, and unsupportive workplace environments. Survivors require psychosocial, financial, and employer support to manage these difficulties. This paper recommends a multiprong approach, including education programmes (facilitated through workers’ union groups, human resource institutions, and/or large consumer CRC groups) and policies, to support workers and for employers to understand the unique challenges of employees with CRC-A.

**Supplementary information:**

The online version contains supplementary material available at 10.1007/s00520-022-07307-9.

Colorectal cancer (CRC) is the third-most common cancer worldwide [1]. Approximately 18% of CRC diagnoses are stage 4, while 30–40% of patients who receive curative treatment develop a recurrence [2, 3]. While people with advanced CRC (CRC-A) were previously only treated palliatively, recent advances have enabled potentially curative treatment for CRC-A [1, 4, 5]. Surgical treatments (including pelvic exenteration (PEx) for locally advanced/recurrent rectal cancer, liver resection for liver metastases, and cytoreductive surgery and hyperthermic intraperitoneal chemotherapy (CRS-HIPEC) for peritoneal metastases), with or without adjuvant chemotherapy, can increase median overall survival to between 13.7–63 months [4, 6–13]. Where curative surgery cannot be achieved, palliative chemotherapy can increase average life expectancy from 5–12 months to > 24 months, alleviate symptoms, and improve quality of life (QoL) [14–16]. Thus, continuing employment or returning to work (RTW), which are both personally meaningful and financially necessary for many survivors [17], is now a realistic option for survivors of CRC-A.

Approximately 18% of Australians with CRC are of working age (15–64 years) [18]; those working average 34.6 hours per week [19]. However, 45.8% of Australian CRC survivors are unemployed, versus 29.4% of Australians without cancer [19]. Having had cancer treatment within the past month or being 5 or more years since CRC diagnosis increases survivors’ likelihood of being out of work [19]. Additionally, 43.5% of retired CRC survivors cite ill health as the cause of retirement compared to 28.9% of Australians without cancer [19].

Employment experiences in people with CRC-A are under-studied [20], despite approximately a quarter of < 65-year-olds diagnosed with CRC having stage 4 disease [2]. A recent meta-review noted many survivors face barriers to RTW including physical symptoms, unrealistic employer expectations, and unsupportive workplace cultures; however, most included papers focused on breast cancer [17]. Furthermore, the impact of different CRC-A treatment types (each with unique side effects) on RTW and financial wellbeing is unknown.

To provide a rich in-depth account of this under-explored area, this study aimed to qualitatively explore and compare the different RTW and financial experiences of people who have been treated for CRC-A through PEx, liver resection, CRS-HIPEC, and/or palliative chemotherapy. This study aimed to address the research question: “How does the experience of CRC-A and its treatment impact survivors’ financial wellbeing and ability to continue or RTW?”.

## Method

The Ethics Review Committee (RPAH Zone) of the Sydney Local Health District provided ethics approval for this project (protocol number X20-0028). This study is part of the larger Qualitative Advanced Colorectal Cancer (QuAd-CRC) project exploring experiences of survivors with CRC-A [21].

### Design

This study is a cross-sectional descriptive qualitative study which employed thematic analysis using a framework methods approach. Qualitative semi-structured interview data was supplemented by quantitative demographic, clinical, and QoL data to characterise the sample and inform subgroup analyses.

### Participants

Clinicians from two major hospitals in New South Wales, Australia, identified patients who were: aged 18 years or older; treated for CRC through PEx, liver resection, CRS-HIPEC, and/or palliative chemotherapy; between 0.5–2 years post-surgery or 0.5–2 years post-diagnosis of CRC-A if receiving palliative chemotherapy; well enough to complete study requirements; adequate English speakers; and able to provide informed consent.

Participants were purposively sampled to include equal numbers of people who underwent different treatment procedures. We also recruited for diversity in age, gender, socio-economic status, and time since surgery/diagnosis. Recruitment continued until thematic saturation (no new themes emerging after three consecutive interviews) [22].

Clinicians invited eligible patients to the study via in-clinic follow-up/treatment, mailed recruitment letters, or telephone. Researchers contacted interested participants and sent study materials to those who consented.

### Data collection

After providing online or written consent, participants completed three patient-reported outcome measures (PROMs), either through an online survey platform, or on paper (and returned via mail). These PROMs assessed the following: *QoL* via the Functional Assessment of Cancer Therapy – Colorectal (FACT-C) [23], scored between 0 and 136 (136 = best QoL); *emotional distress* via the Distress thermometer [24], scored between 0 and 10 (10 = most distress; ≥ 5 = clinical levels of distress); and *financial toxicity* via the Comprehensive Score for Financial Toxicity (COST) measure [25], scored between 0 and 44 (44 = best financial wellbeing).

Demographic (age, gender, postcode, ethnicity, education, marital status, number of people in household, employment status, profession, income, and health cover) and clinical (date of first CRC diagnosis, stage at diagnosis, location of tumour (colon/rectum), status of tumour (metastasised/recurrent), date of recurring cancer, number and sites of recurrences, treatments received, treatment date, presence of stoma, and other chronic health conditions) data were collected from participants’ self-report and/or treating clinicians.

A semi-structured telephone interview with a qualitative researcher (CL) was then conducted. Participant consent was verbally re-confirmed at the start of each interview. Questions explored participants’ lives since undergoing surgery/chemotherapy for CRC-A, before focusing on financial and RTW concerns, and specific questionnaire responses (see Supplementary File A). Interviews averaged 67 min (range 35–92). Audio recordings of interviews were transcribed verbatim. Post-interview reflection notes were documented immediately post-interview.

### Data analysis

Demographics, clinical, and quantitative QoL data underwent descriptive analysis.

Qualitative interview data underwent framework analysis [26]: (1) familiarisation with interviews by repeated listening and reflective notetaking; (2) independent coding and iterative discussion of eight transcripts (21%) by CL, RL-P, and PB, to develop a thematic framework; (3) coding all data using a constant comparative approach (where previous data informs future analyses [26]) through NVivo 12 [27], by CL; (4) organisation of all transcripts into a matrix of interview and subthemes, enabling data to be compared by interview or theme; (5) identification, mapping, and interpretation through framework analysis of relationships between themes, participants, and participant characteristics (demographic, clinical, PROMs).

Framework analysis systematically explored thematic differences between patients with different demographics (age, gender, place of residence), clinical characteristics (treatment received, time since treatment), distress/QoL, and financial wellbeing. For distress/QoL, participants were grouped into four groups: (1) low distress, low QoL (*n* = 9); (2) low distress, high QoL (*n* = 17); (3) high distress, low QoL (*n* = 10); (4) high distress, high QoL (*n* = 2). High distress was defined as ≥ 5, as per clinical cut-off levels [24], while low QoL was defined as at or below median (101.5), comparative to other normative standards for the FACT-C [23]. For financial wellbeing, participants were divided into two groups based on their COST scores: low COST (*n* = 10) vs high COST (> 21) (*n* = 28). High COST was defined as greater than the normative median of 21 from previous literature [25]. Rigour was achieved through careful complying with the COREQ checklist for reporting qualitative research [28] (see Supplementary File B).

## Results

### Participants

A total of 38 survivors with CRC-A participated (see Supplementary File C for CONSORT flow diagram of recruitment). The majority (*n* = 22) were female, and median age was 59 years (range 27–84). At time of interview, 9 (24%) were employed full-time, 6 (16%) part-time, 3 (8%) on leave, and 4 (11%) were unemployed or with home duties. Three (8%) were retired before having CRC-A, while 13 (34%) retired after. Seventeen (45%) were from professional backgrounds, and 50% had incomes below $75,000, indicating the sample appears representative of the Australian population, which has an average income of $90,329 [29]. Participants were a median 14 months since surgery (or since CRC-A diagnosis for palliative chemo participants) (see Table 1 for participant demographics and clinical data).

**Table 1:** Participant demographics, clinical data, and PROs (*N* = 38)

Demographics	
Gender, *n* (%)	16 male (42%)
Age, median (range)	59 years (27–84)
Employment status, *n* (%)	
Full time	9 (24%)
Part time	6 (16%)
On leave	3 (8%)
Unemployed/home duties	4 (11%)
Retired	16 (42%)
Profession, *n* (%)	
Professional	17 (45%)
Clerical or administrative worker	4 (11%)
Technician or trade worker	6 (16%)
Manager	6 (16%)
Labourer	3 (8%)
Sales worker	1 (3%)
Home duties	1 (3%)
Education, *n* (%)	
None/primary school	1 (3%)
Intermediate certificate/year 10	7 (18%)
High school completion/year 12	5 (13%)
Technical certificate/diploma	14 (37%)
University degree	11 (29%)
Income, *n* (%)	
Less than $20,000	4 (11%)
$20,000 to $34,999	2 (5%)
$35,000 to $49,999	5 (13%)
$50,000 to $74,999	8 (21%)
$75,000 to $99,999	2 (5%)
$100,000 to $149,999	6 (16%)
$150,000 to $199,999	3 (8%)
$200,000 or above	3 (8%)
Prefer not to say	5 (13%)
Marital status, *n* (%)	
Married/living with partner	20 (53%)
Separated/divorced	8 (21%)
Single (never married)	7 (18%)
Widowed	3 (8%)
Self-identified culture/ethnicity, *n* (%)	
Australian/New Zealand	20 (53%)
UK	9 (24%)
Other European	4 (11%)
South Pacific/Oceania	1 (3%)
East Asian	1 (3%)
South Asian	3 (8%)
Place of residence, *n* (%)	
Metropolitan	26 (68%)
Rural	12 (32%)
Clinical data	
Tumour type, *n* (%)	
Rectal	17 (45%)
Colon	20 (53%)
Bowel, unspecified	1 (3%)
Stage at first diagnosis, *n* (%)	
I	1 (3%)
II	7 (18%)
III	11 (29%)
IV	15 (39%)
Unsure	4 (11%)
Status at diagnosis, *n* (%)	
Recurrent	18 (47%)
Locally advanced or metastatic	20 (53%)
Treatment for advanced CRC, *n* (%)	
Liver resection	9 (24%)
CRS-HIPEC	6 (16%)
Pelvic exenteration	10 (26%)
Liver resection + CRS-HIPEC*	6 (16%)
Palliative chemotherapy	7 (18%)
Additional treatment, *n* (%)	
Previous colorectal surgery	19 (50%)
Hormone replacement therapy	1 (3%)
Adjuvant or neoadjuvant chemo and/or radiation	32 (84%)
Time since treatment in months, median (range)	14 (6–28)
Stoma, *n* (%)	
None	18 (47%)
Temporary, reversed	9 (24%)
Temporary, due for reversal	3 (8%)
Permanent	8 (21%)
Comorbidities, *n* (%)	19 (50%)
PROs	
FACT-C, median (range)	
Liver resection	106 (56–128)
CRS-HIPEC	106 (81–129)
Pelvic exenteration	100 (59–130)
Liver resection + CRS-HIPEC*	104 (96–132)
Palliative chemotherapy	102 (71.7–11)
Distress, median (range)	
Liver resection	4 (0–10)
CRS-HIPEC	2 (0–6.5)
Pelvic exenteration	4 (0–9)
Liver resection + CRS-HIPEC*	3 (0–6)
Palliative chemotherapy	2 (1–7)
COST, median (range)	
Liver resection	30 (10–33)
CRS-HIPEC	28 (21–43)
Pelvic exenteration	37 (4–40)
Liver resection + CRS-HIPEC*	22 (13–44)
Palliative chemotherapy	28 (19–40)

^*^Six participants had received both liver resection and CRS-HIPEC; thus, a fifth combined group was formed

### Quantitative findings

Participants’ COST scores ranged 4–44 with a median of 30, higher than the normative median of 21 [25]. FACT-C scores ranged 56–132 (median = 102) while distress scores ranged 0–10 (median = 3). COST scores were moderately correlated with distress and QoL, such that greater financial wellbeing correlated with lower distress (*r* =  − 0.50) and greater QoL (*r* = 0.44) (see Table 1 for PROs by treatment group).

### Qualitative findings

**Fig. 1:**
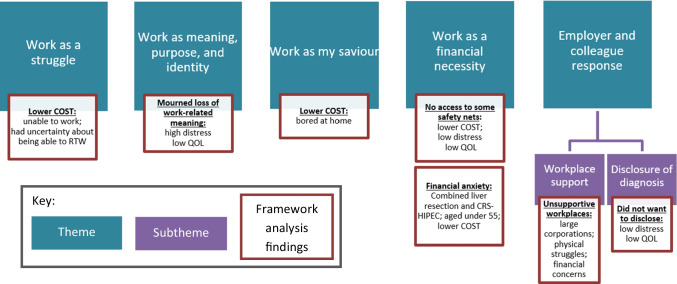
Themes, subthemes, and framework analysis findings

Thematic synthesis revealed five overarching themes (see Fig. 1 for themes, subthemes, and framework analysis findings).

#### Work as a struggle

This theme explores factors that can either contribute to or ameliorate CRC-A survivors’ challenges to continue or RTW. Participants already retired upon diagnosis were not included in this theme. Of the thirteen who retired after diagnosis, six expressed strong desires to RTW. Of these, all had recurrent CRC-A, and four had low QoL.

The most common challenges in working were the physical impacts of CRC and its treatment, particularly chemotherapy side effects, including fatigue, issues with mobility and sitting, pain, and weakness. Indeed, one survivor of palliative chemo (Survivor_PallChemo) stated their “*lack of energy*” meant that continuing work “*was not possible*”. One factor that ameliorated challenges to RTW caused by physical impacts for participants was being able to work from home due to COVID-19. This helped them to RTW while still recovering, which reduced their time off work.

*“Because of COVID… working from home actually allowed me more space and time to recover… I didn’t have to stop work to recover, I was allowed to integrate work and recovery together. (Survivor_LR/CRS-HIPEC)”*

For several participants who would like to RTW, there was uncertainty surrounding when or if they would be able to RTW, due to uncertain treatment duration, prognosis, and future impact of side effects.

*“I’m still on the chemo… it can make you feel sick… once or twice a week you can get a bit of diarrhoea from it… my hands hurt, they go all red, and my feet are red… that’s the side effects I’m getting from the chemo… So, I couldn’t go back to work… Because I’m on my feet for ten, 12 hours a day… My goal is to go back to work but I don’t know what I’m going to do and what aspect of work I’m going to do… it’s the uncertainty that’s the scary bit. (Survivor_CRS-HIPEC)”*

Two survivors, who had physical labour jobs, felt at the mercy of their doctors to determine them fit to RTW, resulting in feelings of helplessness and agitation at the delays and struggles to RTW when they did not receive clearance from their doctors.

*“My doctor… doesn’t want me to go back to the job I was doing… so I’ll have to look for a different type of job… I need to get a letter off him to say I’m fit for work and I don’t know if he’s going to do that for me. (Survivor_CRS-HIPEC)”*

Furthermore, the perceived stigma of bowel, bladder, and stoma bag challenges was a major barrier to RTW, with one survivor of liver resection/CRS-HIPEC (Survivor_LR/CRS-HIPEC) describing her diarrhoea as “*quite embarrassing*”, especially as “*we only have two toilets at work… it’s too stressful to worry about that*”. Additionally, some participants were unable to work around their chemoradiotherapy treatment regimens, with one survivor of PEx (Survivor_PEx) stating, “*the logistics of having radiation every day and working wasn’t going to work… so I quit*”.

Some participants who did RTW still found work a challenge, and either quit work or decreased working hours to reduce work-related stress, with one Survivor_PEx believing “*the stress I’d had contributed to my disease*”. Indeed, some participants who were unable to RTW due to having CRC-A reported positive outcomes from not working, including spending more time with family, focusing on hobbies and recreation, and being less stressed.

While some participants did RTW in the long term, the challenges experienced made it difficult to remain motivated to work or pursue career progression. Other participants had job promotions or retirement directly hindered by CRC-A. Indeed, one Survivor_CRS-HIPEC who developed a recurrence after successfully applying for a senior job position stated “*I couldn’t take [the job]. So, I asked them if they could hold the job for me, but they couldn’t… it was really horrible, and mentally, it really threw me…*”.

#### Work as meaning, purpose, and identity

Some participants mourned the loss of work-related meaning and purpose in their lives, with one Survivor_PallChemo stating, “*I kind of feel useless because I’m not contributing to society in a meaningful way*”. People who expressed such thoughts tended to have high distress, low QoL.

Furthermore, some participants viewed their work as a source of joy and achievement. Indeed, one Survivor_LR/CRS-HIPEC, who had studied throughout recovery to gain professional accreditation, continued working because “*there’s no way I’m going to give that up… it was really bloody hard*”. Another Survivor_LR/CRS-HIPEC stated, “*I’m doing [work] for fun. Just because I had cancer, why stop having fun?*”.

Other survivors, unable to return to their previous longstanding jobs which formed a significant part of their identity, were distressed at the thought of having to develop a new identity in a new work environment.

#### Work as my saviour

This theme revealed the benefits of work for several participants, particularly survivors of surgical treatments, who were well enough to RTW. Some participants worked throughout chemotherapy or radiotherapy treatment, while others waited until they had recovered from treatment.

*“I was working while I was having [chemotherapy and radiation] treatment… I’d go in the morning, have my radiotherapy, go to work, or I would finish work and have radiotherapy… It wasn’t like I had to go to work. It was more, “I am bored. I want to go to work,” …for my sanity. (Survivor_PEx)”*

To some, work was a means of introducing normality and routine, as well as distraction from CRC recovery and fear of cancer recurrence, and to prevent depression. Others described work as “*good for my mental health*” and a means to realise “*my social interaction*”. These sentiments were shared by some participants who were unable to work, stating that with unemployment, participants “*get bored fairly easily*” and lacked opportunities of “*meeting new people*”.

#### Work as a financial necessity

This theme explores the impacts of CRC-A and its treatment on finances and RTW. Most participants did not experience much financial hardship from the direct costs of cancer treatment, but were more indirectly financially affected as CRC impacted their ability to work in the same capacity as before.

Indeed, on the one hand, participants reported that financial safety nets, including the Australian Medicare system and private health insurance, allowed for affordable access to health services. Some participants were eligible for discounted concession, pensioner, or disability rates for medication. Stoma participants praised the Australian Stoma Appliance Scheme, which makes purchasing stomal equipment affordable. Other types of safety nets included having savings, superannuation, or other forms of income such as having their spouse continue working, social security payments, or returns from investments.

However, a minority of participants did not have access to certain safety nets, and thus were financially impacted by high medical costs, which at times led to relationship conflicts. For one Survivor_LR/CRS-HIPEC who “*didn’t have a [private] health fund*”, the “*$20,000 [spent] to have the bowel surgery… more or less shot our marriage*”. Furthermore, many participants experienced financial impacts of CRC-A due to their inability to RTW. This led to financial anxiety and concerns about how to provide for themselves/family.

*“I mightn’t be able to return to work full-stop… I’m nervous about it. I’ve got a young family, a mortgage… it’s stressful… and you just don’t know. (Survivor_CRS-HIPEC)”*

Financial challenges from not working caused flow-on effects, such as limiting socialising, travel plans, and recreational hobbies and exercise.

*“Financially speaking I’m on a very short wick. That’ll be the greatest reason of not being sociable. (Survivor_PEx)”*

One self-described “*painfully independent*” survivor living with family members felt she had lost her financial independence because of not being able to RTW due to CRC, stating that if she were cancer free, “*I think that I would have remained independent, and I would have set different goals for myself*”.

Some participants were also concerned about not leaving a financial legacy for their family after they passed.

*“I might have to sell the house to keep us afloat… I’ve worked hard for that. And that’s what I want to leave for the kids. It’s not about me, it’s about them. (Survivor_CRS-HIPEC)”*

These concerns led one survivor of liver resection (Survivor_LR) to work through his recovery, instead of using up available sick leave. On the other hand, one survivor had used up all her sick leave since “*each time I had operations I’d have two/three months off*”, and was now resorting to unpaid leave whenever she needed to recover from her ongoing palliative chemo treatment.

While some participants did receive income protection through their employment or superannuation, they were uncertain they would be able to RTW once the income protection finished, again resulting in financial anxiety.

Framework analyses revealed that financial anxiety was most prevalent in the combined liver resection/CRS-HIPEC group, as well as participants aged under 55. Financial concerns were present in both married and unmarried (single, separated/divorced, widowed) participants. Furthermore, most participants with lower COST scores were unable to work, had uncertainty about being able to RTW, felt bored at home, did not have access to some safety nets, and experienced financial anxiety. Contrastingly, most with higher COST scores were either retired or had RTW. Finally, there were similar expressions of financial anxiety across genders and distress/QoL scores.

#### Employer and colleague response

This theme explores participants’ perceptions of their employers and colleagues (a key factor in whether RTW was a positive or negative experience), and disclosure of CRC-A diagnosis in the workplace.

##### Workplace support

This theme describes the varying levels of workplace support received by survivors with CRC-A. Several participants stated the importance of employers being “*understanding and flexible*”. One PEx survivor stated employers need to trust and “*believe*” their employees, and offer “*return to work programmes*”. These were important to overcome barriers posed by physical side effects that limited capacity to work, for participants still wanting to RTW.

Some participants experienced a supportive workplace environment that fostered their ability to RTW. Several participants were able to take leave to recover, with employers indicating their jobs would be kept open for when they were well enough to RTW, relieving the stress of finding a new job post-recovery.

A minority of participants’ workplaces had HR policies in place to ensure they received adequate income protection to cover their leave. One workplace “went beyond expectations to help” by gifting a participant food vouchers. For several participants who RTW, their workplaces and employers were “sympathetic”, offering adjustments to accommodate their physical side effects and limitations. These included returning at a part-time capacity before gradually building up to full-time hours.

One survivor’s employer made agreements to accommodate her bowel challenges, which alleviated any concerns of perceived stigma. Another participant’s employer offered to alter her school-teaching duties to accommodate her hand-foot syndrome. Having a supportive workplace environment was typically experienced by people who also viewed work as their saviour.

Unfortunately, a minority of participants experienced a lack of workplace adjustments to accommodate their physical side effects, or a lack of suitable leave. All participants who reported unsupportive workplaces stated it was due to working in large corporations. While participants’ direct managers were supportive, higher-up managers were reportedly not as accommodating, with one Survivor_PEx stating:

*“I quit… it was in a retail store. So there was a head office, and then it was us. So my manager is who I had the good relationship. Head office offered sympathy and empathy, but that was about it.”*

Another shared a similar sentiment:

*“I retired… The place I was working was a very toxic environment… my peers and my immediate general manager were very good. But above that it was very little interest at all. (Survivor_PEx)”*

Workplace support did not appear to influence financial toxicity, as many participants who had lower COST scores also expressed having supportive workplaces. However, all participants who reported having unsupportive workplace environments also had physical struggles associated with RTW and financial concerns.

##### Disclosure of diagnosis

Disclosure of cancer diagnosis was another aspect of work that needed to be navigated.

Many participants felt they could be open about their cancer diagnosis to their employers and colleagues, who they considered friends who “lived the disease” with the participant.

A minority of participants selectively disclosed only to colleagues they were close to or worked directly with, to avoid “people look[ing] at me with sadness or feeling sorry for me” or “being known as the woman with cancer”, highlighting the perceived stigma of having cancer. This was particularly the case if participants worked in larger organisations.

 Participants were grateful when their managers were discrete in disclosing to colleagues. However, a minority of participants had no control over disclosing their cancer diagnosis as the company “had to let people know”. Desire to disclose CRC diagnosis to employers and colleagues was similar between male and female participants, and participants who did not want to disclose tended to have low distress, low QoL scores. Selective disclosure only occurred in participants who reported having a supportive workplace environment (see Table 2 for additional quotes).

**Table 2:** Additional quotes

Subpoint	Example quote
Theme 1: “Work as a struggle”	
Physical impacts of CRC and its treatment make RTW a struggle	“My mobility, that was probably the hardest part about returning to work, I didn’t have mobility.” (Survivor_PEx)
	“I can be somewhere and then I just leak out [from my SPC] and so I choose not to go out unless I really have to… the main part of [my job] is I am travelling a lot and I’m going from site to site… So it’s not like I can sit in the office and go to the toilet every five minutes just to make sure that it’s always empty. I just can’t do that. And as I said I’ve got no control… It is very unpredictable.” (Survivor_PEx)
Working from home due to COVID-19 helped the RTW process	“I didn’t have to be anywhere and you couldn’t go anywhere [because of COVID]. That was good that I could recover. And everyone was home at the same time. …I started to work from home a couple of months before I went back in the office.” (Survivor_LR/CRS-HIPEC)
Uncertainty of when treatment will end, to RTW	“I think my chemo is going to end, probably [in a few months]? …And hopefully I won’t need any other treatment, but… it’s all very much up in the air.” (Survivor_LR/CRS-HIPEC)
Uncertain if able to RTW	“[RTW] kind of depends upon prognosis as well. I mean the other thing with that is that I have to have chemo every two weeks… So it would be hard to imagine what sort of job I could do… apart from running my own business, I don’t really have any qualifications in anything so I wouldn't know where to begin.” (Survivor_PallChemo)
	“I did think of going back to work, though. But, my concern is will my body [handle] all the stress jobs? …That’s why I have doubt [about] go[ing] back to work.” (Survivor_LR/CRS-HIPEC)
	“I think that I could transition to retirement… or I may well choose to go back full time. It’s dependent on how I go… This [cancer] could become aggressive, this could travel to a part of my body that they can’t treat or manage as well. You know, it can change very, very quickly. So I don’t know what the future is.” (Survivor_PallChemo)
Feeling helpless and agitated when not able to RTW without doctor’s clearance	“When I started chemo after the operation… it took me near on a month to get an answer out of the doctor as to when I could possibly return to work so that I could claim for income protection. But they just weren’t really giving me a timeframe to return, based on what sort of a reaction I’m going to have with the chemotherapy.” (Survivor_PEx)
RTW was challenging and stressful, leading to reducing work load or quitting	“My job’s very stressful and I am cutting back.” (Survivor_LR)
	“Because I quit my job, I made my life easier, so it’s not as tough as it seems… I don’t have much pressure.” (Survivor_LR/CRS-HIPEC)
Work was a struggle, and there are benefits to not working anymore	“[Not working]’s good for me. My garden was ignored for a year, so I’ve been spending a lot of time bringing that back. I’ve also started painting the inside of my house, freshening it up. So I’m really enjoying not working and I’m enjoying having days with my son.” (Survivor_PEx)
Hard to work when feeling like they have lost motivation	“I just lost little bit [of] motivation. I do work, it’s not an issue, but I just have to push myself in my head, “OK, you go back to work.”” (Survivor_LR)
	“When you finish University… you want to get promoted and try new skills… But [CRC-A] was a little bit of a setback for me… I’ll still do my job and I still do a bit more challenging work towards the career development but not as much [as] how focussed I was before.” (Survivor_CRS-HIPEC)
Retirement or promotion delayed due to cancer	“[Cancer]’s just slowed [my future work plans and goals] down a bit. It’s probably put an extra year or two on my retirement… I’ll probably stay [working] a bit longer than I was going to.” (Survivor_PEx)
	“Just before I found out I had the cancer, I applied for a job… and it was the [senior] position, which I did get. And then I couldn’t take it. So, I asked them if they could hold the job for me, but they couldn’t… it was really horrible, and mentally, it really, really threw me…” (Survivor_CRS-HIPEC)
Theme 2: “Work as meaning, purpose, and identity”	
Mourning the loss of work-related meaning, purpose, and identity	“I’m 200 per cent dedicated to work when I work… That’s what I miss.” (Survivor_PallChemo)
	“I think it’s good for everybody to get back into things and get a little bit of confidence back rather than just sitting at home seven days a week, not really interacting, I think it’s good for your self-esteem and get rid of that “Mummy” tag and become [myself] again.” (Survivor_LR)
Unable to return to previous longstanding job, which formed a significant part of their identity	“I was a [tradesperson] for 20-odd years, and then I went into supervision… So, with [my doctor] saying [I can’t return to work], he’s thrown a spanner into the works, so now I have to look at something inside which I’ve never done… it’s the uncertainty that’s the scary bit. I’ve worked all my life.” (Survivor_CRS-HIPEC)
	“I tried for so many years to get into [this job], and then when I got there… I had the perfect life… getting sick has just wrecked all those plans for me now… I want to… get back to work… I don’t know what path I could choose, but I definitely can’t go back to [this job].” (Survivor_LR)
Theme 3: “Work as my saviour”	
Work is good for mental wellbeing	“Work for me is good for my mental health. I’m actually better… doing things than sitting at home… so I was happy to be back at work and I’ve got an incredibly supportive staff who know me through both my cancers and [the clients]… they take your mind off things pretty quickly so I didn’t have time to dwell on things.” (Survivor_CRS-HIPEC)
	“Work, to me, was a bit of a saviour… it helped me… Getting out of the house, because I think the more time you spend in the house and alone… that can get into depression, which I didn’t want to go down. And there’s eight of us [colleagues] and they’re friends. And it was my social interaction, coming to the office.” (Survivor_CRS-HIPEC)
Working for normality and routine	“It wasn’t like I had to go to work. It was more that, “I am bored. I want to go to work and there’s nothing wrong with me… for my sanity. Yes, “I’ve had enough of home.”” (PE survivor)
	“Six months [of chemo were] gone and [I] started recovering… so [I] decided to [go] back to work to get some sort of normal day-to-day routine life.” (Survivor_CRS-HIPEC)
Unemployment as boring	“[Not working] drives me f***ing nuts… I’m climbing the f***ing walls around here… the hardest thing I think I’ve ever had to do is do nothing.” (Survivor_PEx)
	“When I was running my own business, so I’m up and out every day, and it’s no big deal to have meetings with people or go and have a coffee or something nearly every day with somebody… And that doesn’t happen anymore.” (Survivor_PallChemo)
	“I like to be doing something and I get bored fairly easily. One can only read so many books, do so many crosswords and things. I enjoyed working.” (Survivor_PallChemo)
Theme 4: “Work as a financial necessity”	
Did not experience much financial hardship from direct costs of treatment, due to Australian Government and Medicare system	“Medicare covers a lot, but I do have private health and that covers the majority of what’s left. So no financial impact on me at all.” (Survivor_LR/CRS-HIPEC)
	“I think [the] Australian Government is really good, I did not spend much on the medical. I had a colonoscopy last week, and it’s free! Almost everything is free.” (Survivor_LR/CRS-HIPEC)
	“I was travelling about 200 kms a day to get my treatment. So, [the Government] reimbursed me… they put about $200/$300 in my account to cover my fuel… And they paid for my accommodation…when I was getting [my] radiation treatment.” (Survivor_PEx)
Discounted medication and stomas	“For some medications, it was only $6.30.” (Survivor_PallChemo)
	“The appliances and the stuff I get [for my stoma]… it’s government subsidised, I’m not actually paying for that… I just pay for the postage.” (Survivor_PEx)
Concerned about financially providing for family	“It’s the lack of the financial security. Because you have no income…I got two kids to raise.” (Survivor_LR/CRS-HIPEC)
	“I hardly ever call in sick… because I was thinking about my wife, my family and how they would do financially if I wasn’t there, so I tried to minimise how much leave I would take so that if anything we would be able to use it as a last resort… I’m the biggest earner in the family so if something was to happen to me then that would be a bit of an issue.” (Survivor of liver resection [Survivor_LR])
Financial anxiety from uncertainty about RTW after income protection ends	“That’s a financial burden because I’m the breadwinner of the family… I really don’t know what’s going to happen in the future. I know that I’m only covered up to the age of 67 but I’m hoping that I'm well and truly back to work by then.” (Survivor_PEx)
Financial challenges limiting social activities and travel plans	“I used to do aqua very regularly… and I absolutely love it. I had to defer my membership because it wasn’t economical.” (Survivor_PallChemo)
	“We don’t have the luxuries that we used to. And because we don’t have family here, flying [interstate] to see them, it’s really expensive for all four of us. It costs over $1000 in flights. And we can’t just say, “Let’s just go up for the weekend,” so I certainly miss that.” (Survivor_PEx)
Theme 5: “Employer and colleague response”	
Subtheme 5A: “Workplace support”	
Supportive workplace	“I’m a schoolteacher so I took a whole term off work but I returned back to work… that year, full time.” (Survivor_CRS-HIPEC)
	“I was on sick leave… but… my job is still there for me when I want to go back.” (Survivor_CRS-HIPEC)
	“They gave me twelve months… to make a decision whether I wanted to come back to work and, in what capacity… so they’ve been absolutely fantastic.” (Survivor_PEx)
Supportive HR policies and RTW programmes	“[My HR person] said, “The company has an insurance policy, income replacement,” …So for the first 18 months I didn’t even have to use my sick leave [or] my super.” (Survivor_LR/CRS-HIPEC)
	“I went back to work for about a year, part time, slowly easing into it, I was doing two days a week, and then three days a week and occasionally four if we were busy.” (Survivor_CRS-HIPEC)
Workplace accommodations	“We’ve got an agreement at work that whenever I feel like it, I just walk out and go home; luckily I only live ten minutes down the road… and go to the toilet… it took the stress away. Because the biggest problem is the toilet.” (Survivor_LR/CRS-HIPEC)
	“I didn’t have playground duties… they took me off to try and get me off my feet… so work was very understanding.” (Survivor_CRS-HIPEC)
Unsupportive workplace	“The Enterprise Agreement says that once you exhaust all of your entitlements, they give you 18 weeks to return back to work on full duties, and if you don’t… the company can put you off… I exhausted all my leave and then the 18 weeks. And basically, that was it. Done and dusted.” (Survivor_LR)
Large organisations as unsupportive	“They’ve got no obligation to look after me financially… A multi-million-dollar company.” (Survivor_CRS-HIPEC)
Subtheme 5B: “Disclosure of diagnosis”	
Openly disclosed CRC-A diagnosis to workplace	“I was happy to be back at work and I’ve got an incredibly supportive staff who know me through both my cancers.” (Survivor_CRS-HIPEC)
Colleagues were supportive in response to cancer diagnosis	“My workmates… they’re my friends as well so they were very supportive.” (Survivor_CRS-HIPEC)
	“My staff lived the disease with me… they spent more time with me in the final parts of recovery probably than my wife did. She only put up with me when I’m home, but they were putting up with me all day.” (Survivor_PEx)
Selectively disclosed diagnosis to few people in workplace	“I didn’t really let anyone know until the very last moment.” (Survivor_CRS-HIPEC)
	“[I was] as open as I wanted to be. I didn’t tell everyone what happened to me, and they still don’t know.” (Survivor_LR)
	“When I left, a few people knew that I’m sick but my manager obviously kept it private. He just shared with what he had to share with, like directors or senior management, those people, HR.” (Survivor_CRS-HIPEC)
	“Some people know that I was sick but they don’t know what I had or to what extent… (Survivor_CRS-HIPEC)
No control over disclosure in workplace	“The company – I didn’t want anyone to know but they had to let people know.” (Survivor_CRS-HIPEC)
	“I don’t even know who has how much information [about my diagnosis].” (Survivor_CRS-HIPEC)

## Discussion

This study explored and compared the RTW and financial experiences of people treated for CRC-A through PEx, liver resection, CRS-HIPEC, and palliative chemotherapy.

We found that many survivors with CRC-A desire, and have the capacity, to continue working after treatment. For many CRC-A survivors, working had perceived benefits, including strengthening their sense of identity, improving mental wellbeing, and providing financial stability.

While many cancer survivors share this desire to RTW [17], survivors with CRC-A face unique challenges (including bowel dysfunction or chemotherapy-induced peripheral neuropathy) that may hinder RTW, with negative emotional and financial consequences. Indeed, participants who were unable to RTW tended to have lower COST (financial wellbeing) scores.

Participants discussed the importance of a supportive work environment, especially when managing stigma-related challenges (e.g. navigating toilet access in their office). For some, the compounded embarrassment and stigma of toileting issues in addition to the cancer diagnosis [30–33] greatly influenced their decision to cease working.

Survivors with CRC-A working in large corporations felt particularly unsupported, with inflexible policies resulting in loss of employment for the survivor. Flexible policies tailored to employees’ individual needs would improve such outcomes. For example, in physical jobs, where side effects may have greater impact [34], finding alternative work for survivors may be required.

With increased working from home during the COVID-19 pandemic [35], some of these challenges may be ameliorated. Research suggests that working from home appears beneficial for people with disability [36]. Further research should explore the perceptions of people with CRC-A, and their employers, of working from home arrangements.

This study highlights the importance of educating employers to understand the specific needs of employees with CRC-A. While resources on RTW and cancer are provided by Cancer Council Australia and the UK’s Macmillan Cancer Support [37–40], none specifically focuses on CRC-A survivors. Additionally, cancer survivors, employers, and clinicians may be unaware of these resources. Further research is required to develop CRC-A-specific resources, and determine how best to disseminate them to employers, e.g. through workers’ union groups, human resources institutions, and/or large consumer CRC groups. Furthermore, a systematic review on interventions to enhance RTW for cancer patients [41] suggests that multiprong approaches are required to support cancer survivors to RTW, including physical, psycho-educational, and occupational aspects. Thus, systemic changes to insurance and welfare policies may also be required to alleviate CRC-A survivors’ financial concerns [41].

Many survivors unable to RTW reported higher distress and lower QoL, and noted financial anxiety and loss of deriving meaning through work, suggesting that additional support is required for this group. A recent review of psychosocial interventions found that meaning-based psychological interventions improved meaning and QoL in advanced cancer survivors [42], while cognitive behavioural therapy (CBT) helped patients manage specific concerns such as symptoms [42]. Further research is needed to examine the effectiveness of meaning-based and CBT-based interventions for survivors with CRC-A in the work context.

When survivors with CRC-A RTW or continue working, they must navigate disclosure of their cancer within their workplace. Our participants whose cancer diagnoses were unwillingly disclosed tended to have low QoL. Furthermore, concerns about disclosure may impact survivors’ desire to RTW. Information about sensitive disclosure and cancer survivors’ privacy rights are less focused on in Cancer Council Australia and Macmillan Cancer Support resources addressed to employers as they are in those addressed to survivors [37–40]; this may be an area needing revision.

We also found that RTW was at times a financial necessity, and financial toxicity appeared to be associated with higher distress and lower QoL. While financial literacy was not assessed in this study, only 33% of all adults worldwide are financially literate [43]. Financial education programmes for CRC survivors and their families may be beneficial, perhaps facilitated by large consumer CRC organisations.

This study’s strengths are its inclusion of patients receiving different treatments for CRC-A, and triangulation of both qualitative (interview) and quantitative (PROMs) data using rigorous framework analysis methodology.

Participants were recruited across two Australian hospitals; however, most participants interacted with multiple hospitals and doctors due to their complex cancer history, widening the applicability of findings. While a diverse sample was sought, most participants were Caucasian, tertiary educated, and from a professional background. Future research should be inclusive of survivors from culturally and linguistically diverse backgrounds, blue-collar industries, or with less education, as these survivors may have unique challenges. Further research is required to examine the generalisability of findings in a larger quantitative study.

Overall, the current study has provided new insights into the experiences of RTW and the financial challenges of people with CRC-A, and has identified areas where workplaces and consumer CRC organisations can better support survivors with CRC-A.

## Supplementary information

Below is the link to the electronic supplementary material.

Supplementary file1 (PDF 77 KB)

Supplementary file2 (PDF 116 KB)

Supplementary file3 (PDF 79 KB)

## Data Availability

The data that support the findings of this study are available from the corresponding author upon reasonable request.
