# Supplementary material for: Work: saviour or struggle? A qualitative study examining employment and finances in colorectal cancer survivors living with advanced cancer
Source: Support Care Cancer. 2022 Aug 16;30(11):9057–69. doi: 10.1007/s00520-022-07307-9 (PMC9378257; doi:10.1007/s00520-022-07307-9)
Supplement: Supplementary file 1 — Supplementary file1 (PDF 77 KB) [file 520_2022_7307_MOESM1_ESM.pdf]

Work: Saviour or struggle? A qualitative study examining employment and finances in colorectal cancer survivors living with advanced cancer

Supportive Care in Cancer

Chloe Yi Shing Lim, Rebekah C. Laidsaar-Powell, Jane M. Young, Daniel Steffens, Bogda Koczwara, Yuehan Zhang, The advanced-CRC survivorship authorship group, Phyllis Butow

Corresponding author: Chloe Lim; Centre for Medical Psychology and Evidence-Based Decision-Making (CeMPED), School of Psychology, Faculty of Science, The University of Sydney, Sydney, NSW, Australia; chloe.lim@sydney.edu.au

Supplementary File A. Sample interview questions

Note: main interview questions are labelled with numbers (1, 2, etc.) while prompts and probing questions are labelled with letters (a, b, etc.). More prompts and probes may have been asked depending on the participants' responses.

1. Firstly, how has life been like for you since (your PE surgery / liver resection / CRS-HIPEC peritonectomy surgery / starting chemo)?

Returning to work

2. Prior to the coronavirus situation (around February/March 2020), did cancer affect your ability to work?
3. [If they stopped working]: What stopped you from going back to work (or working your usual hours)?
    a. Did you try to negotiate around this?
    b. How do you feel about not working? Benefits vs downsides
    c. Has not working impacted on your financial situation?
    d. If COVID is a factor: Assuming COVID is over, how do you feel about returning to work?
4. [If they returned to work]: Why did you want to return to work? Why was it important to you?
    a. What made it harder (or easier) for you to return to work?
    b. How did you try to manage these return to work challenges? Did you have to make any adjustments?
    c. How hard/easy was it to make those adjustments?
    d. Do you think you will stay at this job?
    e. Do you think you will stay working? How long? Why?
5. Has cancer impacted your future work plans/goals/opportunities?
    a. How do you feel about that?

Employer and colleagues

6. Do you feel like you could be open about your colorectal cancer with your employer or colleagues?
    a. And ongoing issues?
7. Thinking not only about your own employer but about employers in general, what is the most important thing for employers to do to support their employees who have had cancer?
    a. Did your employer do those things to support you?
    b. Is there anything else you yourself would have liked from your employer that would have helped your return/staying to work?
    c. Is there anything that you think would have helped your employer to support your return/stay at work? (e.g., government)
8. How did your colleagues react to your cancer diagnosis?
    a. Were they helpful or not in your return/stay at work?
    b. Is there anything that would have helped your colleagues to better support your return/stay at work?

Finances

9. Can you tell me about any costs you have had since your cancer diagnosis?
    a. Are there any cancer-related expenses you are still paying for now?
    b. Were these costs manageable for you?
10. How do you feel about these costs?
    a. Did these costs prevent you from doing or buying other things because you could no longer afford them?
    b. Can you think of anything that would have helped you better manage these costs?
