# Supplementary material for: Work: saviour or struggle? A qualitative study examining employment and finances in colorectal cancer survivors living with advanced cancer
Source: Support Care Cancer. 2022 Aug 16;30(11):9057–69. doi: 10.1007/s00520-022-07307-9 (PMC9378257; doi:10.1007/s00520-022-07307-9)
Supplement: Supplementary file 2 — Supplementary file2 (PDF 116 KB) [file 520_2022_7307_MOESM2_ESM.pdf]

Work: Saviour or struggle? A qualitative study examining employment and finances in colorectal cancer survivors living with advanced cancer

Supportive Care in Cancer

Chloe Yi Shing Lim, Rebekah C. Laidsaar-Powell, Jane M. Young, Daniel Steffens, Bogda Koczwara, Yuehan Zhang, The advanced-CRC survivorship authorship group, Phyllis Butow

Corresponding author: Chloe Lim; Centre for Medical Psychology and Evidence-Based Decision-Making (CeMPED), School of Psychology, Faculty of Science, The University of Sydney, Sydney, NSW, Australia; chloe.lim@sydney.edu.au

Supplementary File B. Evaluating the current study against the Consolidated Criteria for Reporting Qualitative Research checklist (COREQ)

**Item**	**Question/topic**	**Comment** (page numbers from Manuscript file)
**Domain 1: Research team and reflexivity**		
	**Personal Characteristics**	
1	Interviewer/facilitator<br>Which author/s conducted the interview or focus group?	CL conducted all interviews (page 7)
2	Credentials<br>What were the researcher's credentials? E.g. PhD, MD	CL is a Psychology PhD student trained in qualitative research
3	Occupation<br>What was their occupation at the time of the study?	
4	Gender<br>Was the researcher male or female?	CL is female
5	Experience and training<br>What experience or training did the researcher have?	CL completed a qualitative research training workshop, as well as conducted qualitative research for her Honours project prior to the current study
	**Relationship with participants**	
6	Relationship established<br>Was a relationship established prior to study commencement?	After participants expressed interest in participating in the study, the researcher contacted participants to provide further information about the study and to obtain contact details to send the questionnaire to participants. After participants completed the questionnaire, the researcher contacted participants to obtain or confirm demographic and clinical information, and to schedule in a telephone interview. Rapport was built during these two points of contact.

7	Participant knowledge of the interviewer<br>What did the participants know about the researcher? e.g. personal goals, reasons for doing the research	Participants were informed that the study was part of CL's PhD project, and were told in general terms the importance of this research in understanding the experiences and needs of advanced CRC survivors.
8	Interviewer characteristics<br>What characteristics were reported about the interviewer/facilitator? e.g. Bias, assumptions, reasons and interests in the research topic	CL was interested in the return to work and financial impacts of advanced CRC survivors due to her background in researching cancer survivors and quality of life
**Domain 2: study design**		
	**Theoretical framework**	
9	Methodological orientation and theory<br>What methodological orientation was stated to underpin the study? e.g. grounded theory, discourse analysis, ethnography, phenomenology, content analysis	Thematic framework analysis (page 8)
	**Participant selection**	
10	Sampling<br>How were participants selected? e.g. purposive, convenience, consecutive, snowball	Purposive sampling (page 7)
11	Method of approach<br>How were participants approached? e.g. face-to-face, telephone, mail, email	In-clinic recruitment during follow-up or treatment, mailing recruitment letters, and telephoning eligible participants (page 7 and Supplementary File C)
12	Sample size<br>How many participants were in the study?	N = 38 (page 8)
13	Non-participation<br>How many people refused to participate or dropped out? Reasons?	See Supplementary File C for number of people who dropped out and reasons why
	**Setting**	
14	Setting of data collection<br>Where was the data collected? e.g. home, clinic, workplace	Telephone interviews were conducted from a private room, either in an office or home setting
15	Presence of non-participants<br>Was anyone else present besides the participants and researchers?	Two participants had carers present to provide emotional support and help with responding to questions. Only the cancer survivors' words are used in quotations.
16	Description of sample<br>What are the important characteristics of the sample? e.g. demographic data, date	See Participant demographics (page 8-9 and Table 1)

	**Data collection**	
17	Interview guide<br>Were questions, prompts, guides provided by the authors? Was it pilot tested?	See Supplementary File A for interview questions. Interview questions were pilot tested with an advanced CRC consumer representative who previously received PE surgery. Further, the first two interview transcripts were read by RL-P and PB to ensure question phrasing and interviewer style were appropriate
18	Repeat interviews<br>Were repeat interviews carried out? If yes, how many?	All interviews were completed in a single session with no repeat interviews
19	Audio/visual recording<br>Did the research use audio or visual recording to collect the data	All interviews were audio recorded (page 7)
20	Field notes<br>Were field notes made during and/or after the interview or focus group?	Post-interview reflection notes were documented immediately after each interview (page 7)
21	Duration<br>What was the duration of the interviews or focus group?	Interviews lasted on average 67 minutes, ranging from 35 to 92 minutes. (page 7)
22	Data saturation<br>Was data saturation discussed?	Recruitment continued until thematic saturation was reached (page 7)
23	Transcripts returned<br>Were transcripts returned to participants for comment and/or correction?	Transcripts were not returned to participants for correction, to preserve their initial perspectives
**Domain 3: analysis and findings**		
**Data analysis**		
24	Number of data coders<br>How many data coders coded the data?	All interviews were coded by CL. 21% of interviews were checked and discussed by RL-P and PB for inter-rater reliability (page 8)
25	Description of the coding tree<br>Did authors provide a description of the coding tree?	Yes, see Results.
26	Derivation of themes<br>Were themes identified in advance or derived from the data?	Themes were derived from the data as per Stage 2 of framework analysis (page 8)
27	Software<br>What software, if applicable, was used to manage the data?	Microsoft Excel and NVivo 12 (page 8)
28	Participant checking<br>Did participants provide feedback on the findings?	Participants were not asked to provide feedback on the findings, to preserve their initial perspectives

Reporting		
29	Quotations presented<br>Were participant quotations presented to illustrate the themes / findings?<br>Was each quotation identified? e.g. participant number	Yes, see Results and Table 2
30	Data and findings consistent<br>Was there consistency between the data presented and the findings?	Yes, see Results and Table 2
31	Clarity of major themes<br>Were major themes clearly presented in the findings?	Yes, see Results and Table 2
32	Clarity of minor themes<br>Is there a description of diverse cases or discussion of minor themes?	Yes, see Results and Table 2
