# Supplementary material for: Work: saviour or struggle? A qualitative study examining employment and finances in colorectal cancer survivors living with advanced cancer
Source: Support Care Cancer. 2022 Aug 16;30(11):9057–69. doi: 10.1007/s00520-022-07307-9 (PMC9378257; doi:10.1007/s00520-022-07307-9)

Work: Saviour or struggle? A qualitative study examining employment and finances in colorectal cancer survivors living with advanced cancer

Supportive Care in Cancer

Chloe Yi Shing Lim, Rebekah C. Laidsaar-Powell, Jane M. Young, Daniel Steffens, Bogda Koczwara, Yuehan Zhang, The advanced-CRC survivorship authorship group, Phyllis Butow

Corresponding author: Chloe Lim; Centre for Medical Psychology and Evidence-Based Decision-Making (CeMPED), School of Psychology, Faculty of Science, The University of Sydney, Sydney, NSW, Australia; chloe.lim@sydney.edu.au

Supplementary File C. CONSORT flow diagram

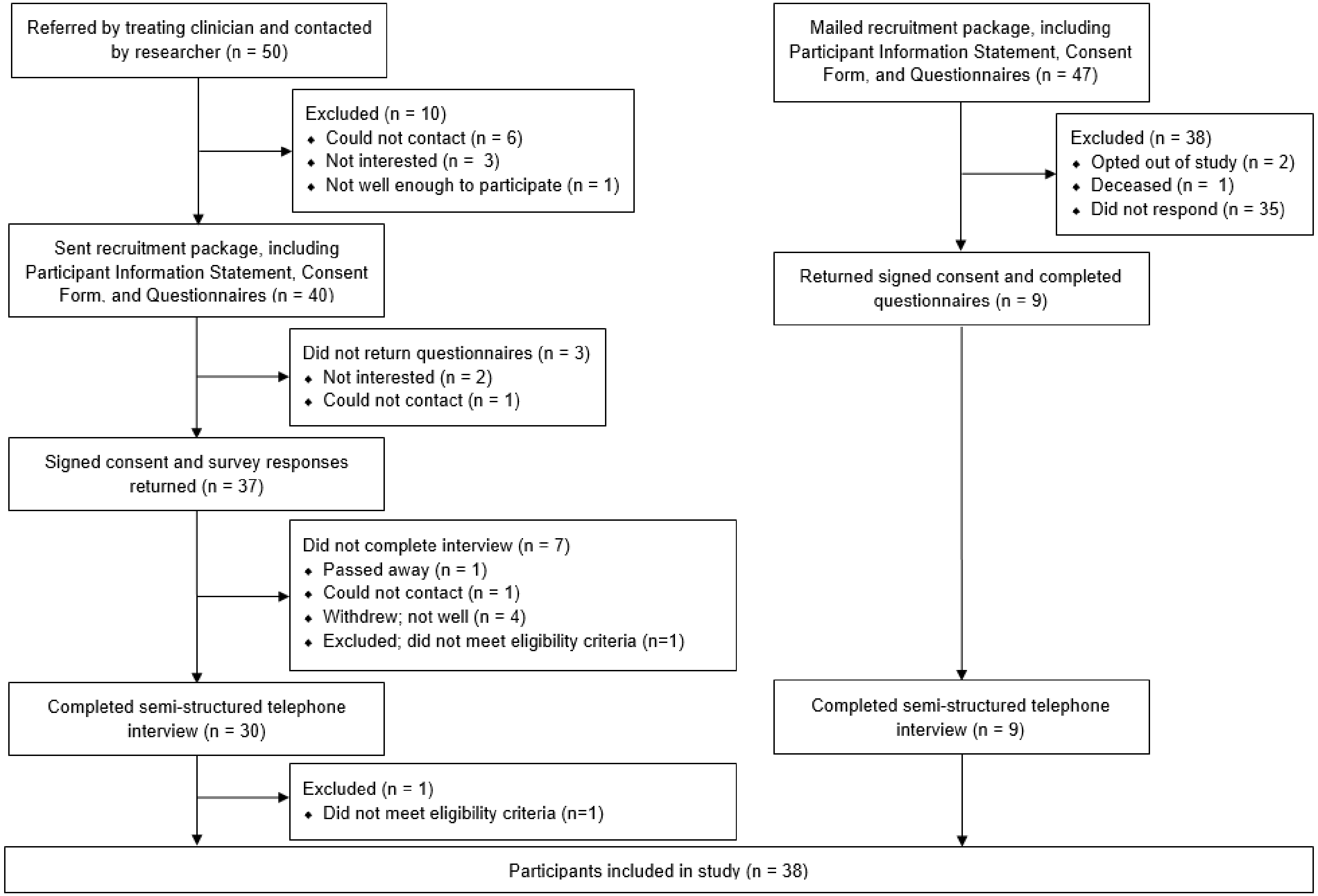

Supplement: Supplementary file 3 — Supplementary file3 (PDF 79 KB) [file 520_2022_7307_MOESM3_ESM.pdf]
